# Industry views about the Banned Drinker Register in the Northern Territory: Early lessons from a qualitative evaluation

**DOI:** 10.1111/dar.13174

**Published:** 2020-09-24

**Authors:** Elizabeth Adamson, Sarah Clifford, Tessa Wallace, James A. Smith

**Affiliations:** ^1^ Menzies School of Health Research Darwin Australia

**Keywords:** Banned Drinker Register, alcohol harm reduction, alcohol policy, Northern Territory

## Abstract

**Introduction and Aims:**

The Northern Territory Government has recently planned and implemented an extensive suite of alcohol harm minimisation policies, including the reintroduction of the Banned Drinker Register (BDR). It is an explicit alcohol supply reduction measure that places persons who consume alcohol at harmful levels onto a register, prohibiting the purchase of alcohol from take‐away liquor outlets. This paper explores industry stakeholders' perspectives regarding the extent to which the BDR is meeting its objectives to improve community health and safety by reducing alcohol‐related harms.

**Design and Methods:**

Interviews and one focus group were conducted with 66 alcohol industry stakeholders from urban and remote locations. Focusing on outcomes both central (crime and safety) and peripheral (health and therapeutic support) to the stakeholders' interest, the authors used inductive thematic analysis to examine participants' perceptions about the effectiveness of the BDR.

**Results:**

Analysis revealed mixed views about the effectiveness of the BDR. There is a tension between the objective to address public amenity and decrease crime, as expressed by the participants, compared to the health‐focused approach to therapeutic services and referrals identified in other sources.

**Discussion and Conclusions:**

Drawing on these findings, alongside other relevant sources, the authors argue there is a need for a more effective communication strategy to the public and professional community to enhance the capacity of the BDR to meet its goals. The authors recognise the limitations of alcohol industry stakeholder views and identify the need for a comprehensive evaluation approach that includes multiple stakeholder perspectives.

## Introduction

The Northern Territory (NT) population has a unique association with alcohol. While many people consume alcohol at low‐risk levels, the NT maintains the highest rates of alcohol consumption in Australia, and similarly high rates of alcohol‐fuelled violence and crime [1]. The social and economic costs and harms of alcohol consumption in the NT are significant and require a strong and sustained alcohol harm minimisation response [2, 3, 4, 5]. This is consistent with the many government‐led alcohol policy interventions in the NT [5, 6]. There are multiple government, non‐government and industry stakeholder groups invested in alcohol reform initiatives. One such policy is the Banned Drinker Register (BDR).

### *Reintroduction of the Banned Drinker Register*

Following the 2017 election of the majority Labor Government, and in response to an NT Alcohol Policy and Legislation Review [1], the Northern Territory Government (NTG) planned and implemented an extensive suite of alcohol harm minimisation policies. Briefly introduced under a Labor Government from 2012 to 2013, then swiftly repealed by the incoming Country Liberal Government, the BDR was reintroduced (by Labor) in September 2017 as part of their electoral commitment. It is an explicit alcohol supply reduction measure that involves placing persons who consume alcohol at harmful levels to themselves or others onto a register, which prohibits the purchase of alcohol from take‐away liquor outlets. The policy is enforced through scanning of valid photo identification for all individuals purchasing alcohol at take‐away outlets in the NT. The information issued to licensees and operators by the BDR software is a simple Yes/No response that indicates if a person is restricted from making the purchase. This occurs immediately prior to purchase. Contrary to some public perceptions, personal details are not stored on the BDR's software. On licensed premises, there are no scanners and the onus is on the banned drinker to adhere to the restriction, rather than the licensee.

The reintroduction of the BDR was designed to be more health‐focused than the previous iteration, including the establishment of a clinical Registrar. Authorised professionals (including: registered nurses; doctors; Australian Health Practitioner Regulation Agency psychologists, psychiatrists, physiotherapists and paramedics; child protection workers; social workers; sobering up shelter team leaders; Public Housing Safety Officers; Aboriginal Health Workers; and Australian Counselling Association Level 4 Counsellors) and family members can refer individuals to the BDR Registrar who determines their placement on the Register and ban length (3, 6 or 12 months). Individuals can also self‐refer onto the BDR. Drawing on policy documentation, as well as interviews and focus groups with Government agencies and industry stakeholders, the 6‐month evaluation of the BDR found the program had been implemented as planned. The evaluation identified multiple areas for improvement, which included addressing secondary supply, promotion of the self‐referral pathway and uptake of therapeutic services [7, 8].

The renewed health focus was intended to better identify and treat alcohol misuse in the NT, through primary health‐care pathways and an increased uptake to voluntarily access treatment services [9]. It is possible this change was influenced by the legal and ethical concerns raised in regard to Alcohol Mandatory Treatment, which preceded the second BDR iteration [10, 11]. Alongside the health‐focus, however, the BDR also explicitly aims to address incidents of crime, antisocial behaviour and community safety [7, 12, 13]. Addressing antisocial behaviour is not a novel aim of NT alcohol policy; Alcohol Mandatory Treatment, a policy implemented between the first and second iteration of the BDR (2013–2017), openly targeted public drunkenness [10]. The ‘Two Kilometre Law’, which has been in place since 1983, and Public Restricted Areas, which were implemented in 2006 [14], similarly aim to reduce alcohol consumption in public places. Police Auxiliary Liquor Inspectors (PALI) were introduced in 2018. PALIs are uniformed constables stationed at take‐away outlets and designed to minimise crime, violence and antisocial behaviour by preventing consumption of alcohol in restricted areas [15]. All of these policies have been criticised as targeting Aboriginal drinkers [14, 16, 17, 18]. The BDR is novel in that it is implemented by take‐away alcohol outlet staff, rather than by police. NTG engaged Menzies School of Health Research to conduct a 12‐month evaluation of the BDR to examine whether it is meeting its overarching goal aimed at ‘*reducing harm to the community caused by alcohol’* [19].

### *What do we know about the effectiveness of individual banning measures?*

Despite the history of local permit systems and bans in the NT in recent decades [17, 20], there is a paucity of research on the effectiveness of individual liquor bans and permit systems. The studies that do exist are primarily of international systems between the 1950s and 1980s, and the results of the effects of individual bans (in isolation) were generally inconclusive [20]. d'Abbs and Crundall [21] distinguish between individual *bans* and *permits*, focusing on the effectiveness of the latter in their review of international and Australian policy measures. Furthermore, research assessing the impact of alcohol reforms and harm‐reduction measures more broadly (i.e. the impact of price controls) has focused on quantitative data to monitor positive and negative impacts, such as the number of hospital admissions, assaults and thefts and crime related to alcohol [22, 23, 24, 25].

Specific to the BDR, available data show that, at June 2019, presentations to emergency departments attributable to alcohol were lower than September 2017; the time the BDR commenced [26]. Other figures indicate that some crimes are decreasing, particularly alcohol‐related assaults (26% decrease) and domestic violence (21% decrease); however, it is difficult to ascertain whether this is a direct result of the BDR, other alcohol‐related initiatives, or other social policies or external factors [22, 27, 28]. As noted by Clifford *et al*. [25], the current NT alcohol policy context is complex and qualitative research is needed to better understand the impact and intersection of local and regional policy interventions, and the context in which they operate.

### *Industry involvement*

The priorities and interests of different groups of stakeholders differ, with a notable tension between commercial interests of industry and public health benefits [29]. Worldwide, the alcohol industry is notorious for presenting itself as socially responsible, and framing harmful consumption as the problem of a small minority not the ‘sensible majority’ [30].

The involvement of take‐away alcohol outlets retailers in this research is somewhat controversial. We posit that these retailers, as the individuals who implement this policy every day, can and do provide a valuable perspective, particularly because they interact with every customer purchasing take‐away alcohol. As implementors, they respond to questions about the purpose and process of the BDR. The majority of the retailers interviewed are also members of their local community, and therefore provide unique perspectives about the perceived impacts of the BDR on public amenity, and community activities and behaviours. In doing so, the paper contributes important findings about the effectiveness of the BDR and fills a significant gap in other, primarily quantitative, data sources that report on the objectives of the BDR [12, 27, 31, 32]. Their views also contribute to a broader alcohol policy evaluation landscape in the NT, such as the minimum unit price [33] and ongoing BDR evaluation work. This paper aims to understand from the perspective of one group of industry stakeholders, the extent to which the BDR is meeting its objectives to improve community health and safety by reducing alcohol‐related harms.

## Methods

This analysis was informed by a realist evaluation perspective [34, 35], which has been adopted in studies of similar alcohol policy interventions in the NT [36]. Using a realist evaluation approach recognises the sociocultural context in which a policy is implemented, specifically the broader, and varied, geographic, demographic and social context in which the intervention and subsequent evaluation takes place. In the context of alcohol policy in the NT, this includes the vast, large distances of the NT, the significant Aboriginal population (25.5% compared to 2.8% nationally [37]) and the intersection of the BDR with other alcohol‐related policies implemented in a similar time period [31, 38]. Unlike program theory evaluation, which assesses whether a program is designed in a way that its intended outcome is achieved, realist evaluation focuses on the real and perceived effectiveness of the policy intervention from the perspective of stakeholders; in this case, the industry stakeholders and take‐away managers and licensees, as detailed below. Industry stakeholders and take‐away managers and licensees' views and experiences provide valuable information about the perceived effectiveness of the BDR within different social, political (including policy), geographic and demographic contexts. Their views alone do not, however, have the capacity to assess the overall effectiveness of the program. In particular, there are key perspectives missing from this analysis, namely that of government stakeholders, health professionals, the general public and people placed on the BDR and their families. Some government stakeholder and health professional perspectives (*n* = 17) are captured elsewhere; broadly these perspectives included the role of BDR in avoiding ‘humbug’ (an Aboriginal English word that refers to exploitative and/or incessant ‘demand sharing’ within family and kinship structures) [39], discussions regarding secondary supply and concerns that Aboriginal people were disproportionality represented on the BDR, considering that both non‐Aboriginal and Aboriginal NT residents consume alcohol in excess of the National Health and Medical Research Council guidelines [13, 40]. Further details of these limitations are discussed in the Discussion.

**Figure A1:**
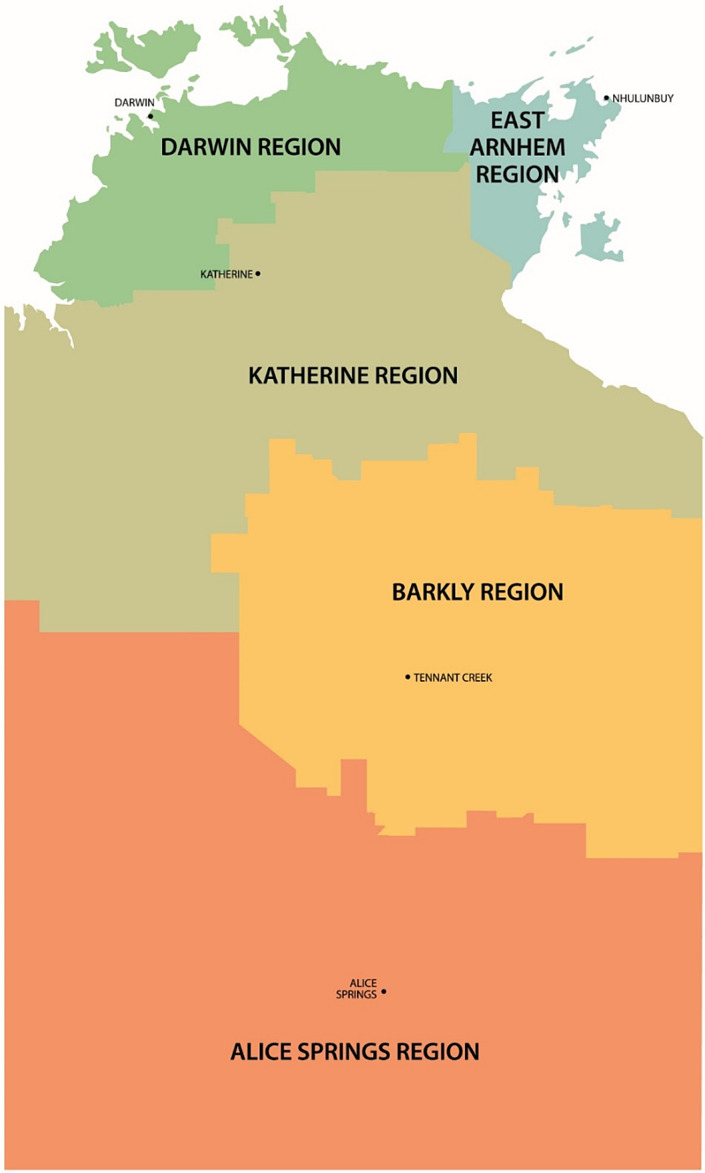
Five regions in the Northern Territory, geographic boundaries.

Qualitative interviews and focus groups were undertaken with industry stakeholders and take‐away bottle shop managers and licensees from urban and remote locations (see Appendix, Figure A1). The interviews were with retailers (bottle shop managers and licensees), and a focus group was undertaken with three participants representing the hospitality industry. The interviews and focus group were undertaken over 3 weeks in May 2019. Using Google searches, the research team identified 116 take‐away outlets across the NT. The research team contacted 97 (84%) of these outlets, specifically those who had clearly identifiable contact information on their website. Of the 97 contacted, 12 formally declined to participate, and a further 34 were unable to be involved. Overall, 51 interviews and one focus group were conducted involving a total of 66 participants (see Table 1). Of the 66 participants, 33 were licensees (or nominated licensees), 30 were managers of take‐away bottle shops and three were representatives of the hospitality industry. Invitations to take‐away bottle shops were directed to the licensee; however, in some instances the licensee passed on the request to the manager or asked if the manager could also participate. Thus, many interviews included two participants from the same bottle shop. Consultation occurred in every region except East Arnhem, because an inclusionary permit system already operates in this region; that is, rather than identifying those who aren't permitted to purchase alcohol, the system identifies those who are permitted to purchase [21]. This research received ethics approval from the NT Department of Health and Menzies School of Health Research Human Research Ethics Committee (2019‐3287) and the Central Australian Human Research Ethics Committee (2019‐3358). All participants provided written or verbal informed consent.

**Table 1:** Distribution of take‐away outlet participants by region

Region	No. of interviews	No. of participants	% of total participants^a^
Darwin region	20	22	35
Katherine region	12	17	27
Barkly region	11	15	24
Alice Springs region	8	9	14

^a^ Excluding the industry focus group (*n* = 3), total number of take‐away outlet participants is 63.

The interviews and focus group were undertaken using a semi‐structured interview schedule (see Appendix S1, Supporting Information), which were audio‐recorded and professionally transcribed. Key themes and findings were identified through an inductive thematic analysis workshop with the evaluation team. The authors used a stepped process of thematic analysis, which involves six stages—familiarising yourself with the data; generating initial codes; searching for themes; reviewing potential themes; defining and naming themes; and producing the report [41]. The research team met face‐to‐face to undertake the first five stages of the analysis; they selected transcripts to review and discussed and identified themes. Further transcripts were then selected to review (with overlap among team members), and the themes were discussed and revised. The themes were then adapted into a coding structure and three of the authors distributed and independently coded the remaining transcripts using NVivo software. Through this process, the authors identified key themes about the perceived effectiveness of the BDR, as presented below.

## Results

### *Perceived effectiveness of the BDR*

#### *Purpose of the BDR and its referral pathways*

Overwhelmingly, licensees and operators perceived the BDR as a crime intervention measure, particularly in regard to drink‐driving and domestic violence. Every participant identified criminal behaviours as the mechanism for receiving a Banned Drinker Order before mentioning other mechanisms (such as referral by a medical professional or family member). For example:

*‘I think the goal is to reduce crime, just have a—it's basically just to reduce* […] *alcohol‐related crime’*. (Participant 40: Katherine, Manager)

Some participants recognised the limited use of self‐referral and health professional pathways and commented on the perceived reasons for their low take‐up. For example:

*‘No‐one really utilises the other part of the BDR. You could put yourself on the Banned Drinker Register but then once they do that then it's like oh well, now I can't buy anything for me when I want to sort of thing, so most people don't take that option’*. (Participant 8: Alice Springs, Manager)

*‘And the demonisation or stigmatisation of putting people on the BDR, they take a harm minimisation approach to distance them. Even though they* [health practitioners] *advocate for it at a high level, they don't want to be the person that actually refers someone onto them’*. (Participant 56: Industry Representative)

Some of the reasons for the low take‐up of some referral pathways, and the potential tensions in the program objectives, are detailed further in the Discussion.

Although the stakeholders did not have specific health expertise, a number of participants commented on the need to shift to a more holistic and health‐focused approach. For example:

*‘There needs to be a better emphasis on how we deal with what people are actually doing as opposed to the punitive throwing them into jail and, it has to be a much, much earlier intervention for people. And it needs to be more health based as opposed to criminal justice*’. (Participant 5: Alice Springs, Licensee)

Participants not only identified a lack of awareness about the BDR among the general population, but also found particular groups lacked knowledge of its purpose. For example, tourists were often confused about why they must present their ID and had unfounded concerns about privacy and collection of personal data (such as identity theft):

*‘I guess there's also a perception that when people purchase alcohol that their purchases are being recorded, so they feel somewhat intimidated and threatened by that. Especially* [those] *from interstate’*. (Participant 5: Darwin, Licensee)

In addition, tourists (as well as taxi drivers) were flagged as two groups of people who potentially facilitated the secondary supply of alcohol to banned drinkers. For example:

*‘Yeah again, they* [people on the BDR] *beat the system, someone else will buy it for them*, […] *a couple of guys were here, and they obviously were on the banned list, and they were literally asking our customers that were driving in, “Can you please go buy us alcohol?”’* (Participant 11: Alice Springs, Licensee)

It was suggested that increased awareness about the risks of supplying alcohol to individuals on the BDR (secondary supply) should also be better communicated across the NT, particularly at border crossings and through tourism information flyers/stations.

#### *Antisocial behaviour, public amenity and crime*

There was little consensus among participants about the impact of the BDR on public amenity, with considerable variation across regions and within urban centres. Public amenity was more often identified as a problem for take‐away outlets in locations with green areas or nearby parks, particularly where groups tended to congregate. One licensee explained this distinction:

*‘Well, I think it's* [BDR] *more effective in town, because they have a lot of long‐grassers* [Note: Predominantly Aboriginal people who set up makeshift camps on secluded vacant land; 42] *and a lot of people that are in that, you know, going to the hospital or to the police station every five minutes* […]*. Whereas we don't really have that sort of demographic. People just buy take away here and go home*. [so] *it's not doing anything out here’*. (Participant 61: Darwin, Licensee)

Changes in public amenity were not generally attributed to the BDR, but rather to local restrictions that led to the reported movement of people to different areas, as well as the lack of enforcement of laws prohibiting drinking in public. However, one participant, located in a small regional town, indicated that public drinking had worsened since the BDR, which they believed was due to police focusing on more serious crime, rather than minor issues, such as drinking in public. For example,

*‘It seems to be that the laws have been dropped for public drinking. I notice that previously they would get their take‐aways and go, and be out of sight. Now it's in the park, in plain sight. Which is against the law, drinking in a public place, but* […] *I think the police have just been told* […] *we're here to fight crime, not pick people up for public drinking’*. (Participant 41: Katherine, Licensee)

In some cases, changes to public amenity and antisocial behaviour were attributed to the impact of the BDR on secondary supply. The effectiveness of the BDR was regularly discussed in relation to the challenges of overcoming secondary supply, which is analysed further elsewhere [13].

Similarly, there were mixed responses about whether the BDR is having a positive or negative impact on crime. Some participants recognised the positive aspects of the BDR as a tool to better implement rules and policies to prevent crime and antisocial behaviour. One manager believed the BDR provided a mechanism to refuse purchases by individuals displaying drunken or anti‐social behaviours:

*‘If an intoxicated person come* [in] *according to the Liquor* [Act], *we can't sell the liquor to him anymore. But if I say to him* [I can't serve him]*, he may go ‐* [wildly gesturing] *‐ something like that… But I show him the BDR, okay, the Government itself is saying that, “Okay, you are not* [allowed to purchase]”*’*. (Participant 17: Alice Springs, Manager)

Others, however, attributed increases in theft and break‐ins of both licensed premises and residences to obtain alcohol to banned drinkers seeking other ways of obtaining alcohol. For example:

*‘Yes, I think there is probably more break‐ins because of it*. […] *People trying to steal alcohol. Probably the same house break‐ins too. You assume people are trying to find grog wherever they can’*. (Participant 47: Darwin, Manager)

Overall, there were mixed perspectives about the impact of the BDR on antisocial behaviour, public amenity and crime.

#### *Use of BDR data*

As noted earlier, the BDR software generates a simple Yes/No response indicating whether a sale can proceed. However, there were significant misconceptions among participants about these nuances, and a number of participants had ideas for how such data could be better collected and utilised. Some participants believed that data about banned drinkers could be better linked with hospital admissions data, information about engagement with other health and social services and crime data.

It was also suggested that a notification system, supported through enhanced data collection, could be used to communicate BDR refusals in real time among licensees, operators, police and outreach health staff within a defined geographical area (e.g. primary trade area of 5 km). For example:

*‘If the BDR worked properly, they could control more. If it recorded where you purchased last so, you know, you've purchased at* [Take‐away Outlet 1]*, and now you're* [at Take‐away Outlet 2] *and I will look at the BDR and say hang on, you've been here, here and here and you're still sober, where's all the alcohol? That's where you then bring in your secondary supply* [measures] *and then the alerts, the police might be able to put an alert on the BDR’*. (Participant 16: Alice Springs, Manager)

Other limitations of the BDR data related to its intersection with other policies (discussed further in the next section), namely the PALIs. A few participants noted that a streamlined or integrated system would be more efficient. For example:

*‘Yeah because you have the PALIs, they do their checks there and then they have different information to what's on the* [BDR] *machine. All the information should be on that machine, then there'd be no reason to have police* [PALIs] *here…’* (Participant 42: Katherine, Licensee)

In summary, there was an appetite from some participants for the BDR IT infrastructure to be used to assist in providing more targeted health interventions to ‘at‐risk’ populations; help to respond to ad hoc social issues impacting public amenity; and provide increased intelligence to curb secondary supply and grog‐running.

#### *Intersection with* *NT* *Alcohol Harm Minimisation Action Plan 2018–2019*

The BDR is one of many recent alcohol harm minimisation strategies implemented by NTG. Participants acknowledged the complexity of NTG's policies and the inability to assess the effectiveness of any measure, particularly in isolation from another:

*‘It's a very complex problem and at least they're* [NTG] *trying. They've got to do multiple strategies. It's not just one answer’*. (Participant 49: Darwin, Licensee)

This was particularly relevant for participants outside the Darwin region, where PALIs play a critical role in regulating access to purchase alcohol. The 2018–2019 Police, Fire and Emergency Services Annual Report notes 34 PALIs are located in Alice Springs, 22 in Katherine and four in Tennant Creek [43]. In these locations, there were several issues raised with respect to the lack of synergy between the PALIs and the BDR. This was generally related to the double‐handling of IDs, as PALIs request ID and may ask subsequent questions (such as ‘where are you drinking?’) upon entry to take‐away outlets. While the PALIs and BDR are different policy measures with different purposes, initially implemented through different Acts, the double‐scanning of IDs reportedly irritated customers and was perceived to be burdensome. As such, there was strong feedback that these processes should be streamlined in an operational sense.

Participants outside the Darwin area frequently reported that people from restricted communities/regions accessed alcohol from other communities/regions where restrictions were not in place, travelling long distances to do so. While it is difficult to validate these claims, they explained that this variation in regional licensing defeated the purpose of local restrictions, concluding that ‘*unless it's streamlined it's not going to work’* (Participant 22, Barkly, Manager). One licensee recommended a community‐led approach whereby restrictions would apply to individuals across the NT:

*‘But I think if communities had the power given back to them to decide, everybody in our community is permitted when they go to town to purchase, and it was linked to the BDR. So every single person in every community was allowed to purchase a six pack a day or a dozen a day or something along those lines there wouldn't be the shift. There wouldn't be the urban shift. There wouldn't be people moving and going all over where they can get larger amounts* [like Darwin]*’*. (Participant 5+6: Alice Springs, Licensees)

These findings emphasise the importance of context in undertaking realist evaluation.

## Discussion

### *Implications for policy and practice*

Findings from this analysis illustrate a nuanced picture of the perceived effectiveness of the BDR is required to assess whether it is meeting its goals. Participants identified complex social and policy interactions impacting the effectiveness, or perceived effectiveness, of the BDR for businesses, and for harmful drinkers, their families and communities. Before discussing the contributions of the findings, it is important to note the limitations of the study, particularly that the findings represent the perspective of alcohol industry stakeholders and do not represent public health professionals, individuals on the BDR, their families or the broader community. We thus acknowledge the commercial interests of industry stakeholders, and the respective power and influence they exert over other stakeholders. That is, managers and licensees of take‐away bottle shops, as well as alcohol industry representatives that participated in the focus group, have an inherent bias and vested interest in the outcomes of the evaluation for the alcohol industry [44]. On occasion, their views and perspectives are at odds with findings and recommendations to introduce alcohol harm reduction strategies. In particular, some literature goes against participants' views about the need to focus on education campaigns [45]. The low number of participants in the focus group is also recognised as a limitation. The voices of other stakeholders, including police, health professionals and policy makers, are equally important to understand, monitor and evaluate alcohol policy development and implementation in the NT. Indeed, a more comprehensive evaluation of the perspectives of key government stakeholders and individuals on the BDR, their families and the broader community will be undertaken longitudinally through an Australian Research Council linkage grant [Learning from Alcohol (Policy) Reforms in the NT: LP180100701], over the following 4 years (2020−2023). Despite these limitations, our findings illustrated that the alcohol industry representatives we interviewed understood how the BDR worked and made clear statements about the impact within the context of their bottleshop and local area. This provided valuable information about the real and perceived effectiveness of the BDR, particularly how perceptions of the impacts of the policy vary across locations.

There is tension between the objective to address public amenity and decrease crime, in contrast to the health‐focused approach to improve access to therapeutic services and referrals by authorised persons. Participants' perceptions about crime prevention as the main purpose of the BDR align with available administrative data, which shows that police and court pathways account for 65.6% and 25.6% of Banned Drinker Orders issued respectively [26], and alcohol‐related contact with the justice system is still the major reason people are on the BDR [12]. Other figures show the low numbers of individuals being placed on the BDR through an authorised person or self‐referral pathways [32]. As other reports have pointed out, participants identified a need for improved community and professional awareness around self‐referral pathways, and referral pathways for authorised professionals [7]. Importantly, in order to better achieve the policy's goal ‘*to improve community health and safety by reducing alcohol‐related harms’*, there must be an increased focus on the barriers to accessing therapeutic supports and services. Participants in the study had mixed views about the effectiveness of the BDR to improve antisocial behaviour, crime and safety. Some licensees and managers believed that the BDR simply shifted anti‐social behaviour to other locations. Miller *et al.'s* [46] review of alcohol harm reduction interventions concluded there is minimal evidence about the effectiveness of individualised controls. This is consistent with Room [20] who points out that such measures could make people feel their privacy is being invaded, or they are being stigmatised or marginalised for their alcohol purchases [46]. Early administrative data suggest that crime and anti‐social behaviour is decreasing [26, 27]; however, it is unclear if this is the result of the BDR, or other alcohol‐reduction/harm‐minimisation and anti‐social behaviour initiatives implemented by the NTG.

Parallel implementation of the minimum unit price is likely to have an impact across the NT [22, 33]. It is also plausible that the PALIs may also have an impact in Katherine, Tennant Creek and Alice Springs. In Darwin, investments in anti‐social behaviour may also have an influence. Anti‐social behaviour investments include increased day patrol vehicles, making it easier to report anti‐social behaviour, and more visible policing to improve the safety in public spaces [25, 47]. There are also other local restrictions that intersect with each of these interventions that should be considered [36]. This will require a combination of quantitative and qualitative evaluation approaches that extend beyond the interview data presented in this article.

Available administrative data that show over 80% of banned drinkers are Aboriginal people [12]. It is thus important to acknowledge the racial undertone to the framing of public consumption of alcohol, which is demonstrated elsewhere [14, 16, 17, 48]. Addressing the structural and systemic impacts of colonisation, racism and intergenerational trauma experienced by Aboriginal and Torres Strait Islander people in the NT and the pervasive and inequitable impacts of the harmful use of alcohol on their health and wellbeing [49, 50] requires more explicit consideration in alcohol policy reforms.

Overall, a number of participants recommended that Government, industry and other stakeholders continue to develop targeted education campaigns to communicate the goals and design of the BDR to the public, as well as the risks of secondary supply. We note that the NT Government [31] has already commenced addressing these concerns; however, it is unclear from current evaluation data whether these actions are working effectively. As it has been found in other realist evaluations of alcohol policy in the NT [36], it is essential that the cultural, social and geographic contexts are considered in any education campaigns and communication strategies. However, education campaigns alone are likely to be insufficient [44, 51]. Similarly, considering intersections between alcohol policy and other health and social policy contexts where inequities are evident is also important [52, 53]. Intersectoral and cross‐government responses that address underlying social determinants of health, which involve engagement with mental health, domestic and family violence, education, housing, justice and child welfare systems, are ultimately likely to have a more significant impact than targeted health education and communication about the BDR. For example, a recent Australian study [54] has looked to international experiences to demonstrate the potential to managed alcohol programs, which are designed to provide a safe and supportive environment for vulnerable homeless populations with alcohol dependence.

## Conclusion

The findings presented in this paper illustrate areas for policy and data improvement. While some participants recommended changes to data collection that would allow for targeted health interventions, this would require significant legislative change and substantial IT infrastructure investment. The challenges of measuring and monitoring the impacts of the BDR are complicated by multiple alcohol‐reduction strategies and restrictions in the NT [13, 25]. It is therefore difficult for stakeholders to know what is driving changes in alcohol consumption, crime and antisocial behaviour. These findings contribute to current gaps in research, particularly in Australia and the NT, about the effectiveness of alcohol harm reduction policies, particularly in relation to banning individuals, IT‐scanning technology, and the need for community‐responsive and targeted education campaigns [36, 46, 55]. As noted earlier in the paper, an important limitation of this study is the absence of other stakeholder groups' views, namely banned drinkers (and their families), health professionals and other Government stakeholders. There is a need for ongoing longitudinal and more comprehensive monitoring, evaluation and research with a broader group of stakeholders.

## Conflict of Interest

The Alcohol and Other Drugs team at Menzies School of Health Research receives funding from the Northern Territory Government.

## Supporting information


**Appendix** **S1:** Interview schedule.
