# Supplementary material for: Industry views about the Banned Drinker Register in the Northern Territory: Early lessons from a qualitative evaluation
Source: Drug Alcohol Rev. 2020 Sep 24;40(2):210–9. doi: 10.1111/dar.13174 (PMC7891360; doi:10.1111/dar.13174)
Supplement: Supplementary file 1 — Appendix S1: Interview schedule. [file DAR-40-210-s001.docx]

## Supplementary: Interview schedule

**Preamble**

1. Ethics
2. Confidentiality
3. Can stop at any time
4. Should take 30 minutes to 1 hour
5. Do you have any questions before we start?

Key research questions include:

1. **Background Information**

- Please tell us a little bit about yourself and your work history. How long have you worked in your current role and setting?
- Were you working as a licensee/manager prior to the BDR?
- Were you working in this space the last time the BDR was implemented? If so, how did this differ to the recent reintroduction of the BDR?
- In your own words, describe what you consider the main goal of the BDR to be about?
- In your own words, please explain who you think the BDR is aimed at and why?

1. **What has happened in the first year of implementation of the BDR?**

- In your opinion, what has worked well (e.g. enablers/opportunities) in relation to the reintroduction of the BDR and why?
- In your opinion, what has not worked (e.g. barriers/challenges) well in relation to the reintroduction of the BDR and why?
- What has the implementation process been like for (a) you, and (b) the business?
- What do you think the public perception of the BDR has been since its reintroduction over a year ago?
- In general, what do you think are the impacts of the BDR? Positive/negative.
- In general, how effective do you think the BDR is in achieving its aim?
- How do you think the BDR will play-out in the future?
- How is the BDR received by interstate travellers?
- If you could change any aspects of the BDR, what would they be and why?

1. **How do banned drinkers react?**
   1. When flashed red – how does the customer handle this? How do you handle this?
   2. Elaborate on any stand-out instances here.

- Who do you think the BDR is targeting? How effective do you think the BDR is in targeting harmful alcohol use?
- Is it capturing the targeted? Elaborate

1. **Secondary Supply**

- Do you think secondary supply is still an issue? Do you think the BDR is effective in addressing the issue of secondary supply? Why/why not?
- Any comments around the secondary supply issue? Elaborate

1. **Crime and Antisocial behaviour**

- Do you think the BDR is affecting crime/antisocial behaviour? Is there less crime, more crime?
- What sort of crime – break-ins/theft, public disturbance, assault/DV, drink driving, underage drinking and drug taking
- Do you think the BDR has reduced the frequency of banned drinkers’ contact with the justice system?

1. **Treatment/Health**

- How do you think the BDR is impacting health of the NT population? Hospital admissions, accidents/incidents, addiction, health and wellbeing. Elaborate
- Did you know people can access specialist therapeutic services as part of being on the BDR?
- Are you aware of any people who have turned to treatment services for support since being on the BDR?
- Do you think more people are using or want to use therapeutic services since the BDR was introduced? Why/Why not?
- Do you think there are any barriers to accessing services? Does the BDR help or hinder this? Elaborate

1. **Other policies**
   1. How do you think the BDR connects with other alcohol-related policies, such as the minimum floor price, PALIs? Elaborate
   2. Do you think the BDR is an effective measure in conjunction with these? If so, in what ways. If not, why not?
   3. Do you have any thoughts about the effectiveness of other alcohol policy initiatives, such as the minimum floor price? (probe: enablers/opportunities and barriers/challenges)
2. **Public Amenity**

- In what ways has the BDR impacted the public amenity around takeaway liquor outlets?
- How has the atmosphere around your premises changed since the BDR? Positive/negative/NA
- How do you think the BDR has affected social interaction in town?
- BDR affected the visibility of drinking and drunken behaviour?
- Any concerns around this? Elaborate.

1. **Wrap-up**

- In the next 12 months with the BDR in place, how do you think the government could improve/address further concerns of alcohol misuse and related issues?
- Do you have any suggested recommendations? Is there anything else you would like to add?
